# Effects of Dangguixu-san in patients with acute lateral ankle sprain: a randomized controlled trial

**DOI:** 10.1186/s13063-021-05135-6

**Published:** 2021-03-04

**Authors:** Jae-Hong Kim, Cham-Kyul Lee, Eun-Yong Lee, Myoung-Rae Cho, Young-Su Lee, Jeong-Soon Lee

**Affiliations:** 1 Department of Acupuncture and Moxibustion Medicine, College of Korean Medicine, DongShin University, Naju City, 58245 Republic of Korea; 2 Department of Acupuncture and Moxibustion Medicine, Semyung University Korean Medicine Hospital in Chungju, Chungju, 27429 Republic of Korea; 3 Department of Korean Internal Medicine, DongShin University Gwangju Korean Medicine Hospital, 141, Wolsan-ro, Nam-gu, Gwangju City, 61619 Republic of Korea; 4 Department of Nursing, Christian College of Nursing, Gwangju City, 61662 Republic of Korea

**Keywords:** Ankle sprain, Dangguixu-san, Herbal medicine, Randomized controlled trial

## Abstract

**Background:**

Dangguixu-san (DS), a herbal extract, is widely used in Korean medicine to treat pain and swelling caused by ankle sprain. However, there is insufficient evidence regarding the effects of DS on ankle sprains. Accordingly, we assessed the efficacy and safety of DS for the treatment of acute lateral ankle sprain (ALAS).

**Methods:**

This study was a multicenter (two Korean hospitals), randomized, double-blind, placebo-controlled, parallel-arm clinical trial with a 1:1 allocation ratio that included a per-protocol analysis and sub-analysis based on symptom severity. Forty-eight participants (*n* = 28 at Semyung University Korean Medicine Hospital in Chungju; *n* = 20 at DongShin University Gwangju Korean Medicine Hospital) with grade I or II ALAS that occurred within 72 h before enrollment were randomized to a DS (*n* = 24) or placebo (*n* = 24) group. Both groups received acupuncture treatment once daily for 5 consecutive days and the trial medication (DS/placebo capsule) three times a day for 7 consecutive days. Primary (visual analog scale [VAS] scores for pain) and secondary (Foot and Ankle Outcome Scores [FAOS], edema, and European Quality of Life Five-Dimension-Five-Level Scale [EQ-5D-5L] scores) outcome measures were recorded at baseline (week 0), the end of the intervention (week 1), and 4 weeks after treatment completion (week 5).

**Results:**

Forty-six participants completed the trial (*n* = 23 each). Changes in VAS scores, FAOS Symptom/Rigidity, and FAOS Ache from week 1 to week 5 showed significant differences between the two groups. Sub-analyses showed significant differences in changes of FAOS Ache (week 0 to week 5) and VAS scores, total FAOS, and EQ-5D-5L scores (week 1 to week 5) between the two subgroups (grade II). There were no adverse events and significant negative changes in clinical laboratory parameters in both groups.

**Conclusions:**

Overall, the results of this study are in favor of DS combined with acupuncture and suggest that DS combined with acupuncture is a safe treatment with positive long-term effects in terms of pain reduction and symptom alleviation in patients with grade I or II ALAS.

**Trial registration:**

Clinical Research Information Service KCT0002374. Registered on July 11, 2017; retrospectively registered.

**Supplementary Information:**

The online version contains supplementary material available at 10.1186/s13063-021-05135-6.

## Background

Lateral ankle sprains are one of the most common injuries experienced during athletic or recreational activities [1], and they are more serious than commonly believed because, in addition to causing immediate-onset pain, swelling, and loss of joint motion, they progress to chronic ankle instability (CAI) in a third of patients [2–4]. CAI is characterized by residual symptoms, including a feeling of “giving way” and instability, recurrent ankle sprains, and functional loss after acute ankle sprains [5]. It not only reduces physical activity (because of persistent ankle pain, stiffness, weakness, and instability) but also leads to the development of posttraumatic ankle osteoarthritis [6, 7]. The high incidence of ankle sprain and the consequent high prevalence of persistent problems increase the burden of chronic health issues in the community [8].

The three major types of treatment for ankle sprain are surgery, conservative treatment involving immobilization with a plaster cast or splint, and functional conservative treatment with bandages, tapes, different braces, and/or balance training [9].

In 2017, ankle sprain was reported as the fifth most common reason for visits to Korean medicine clinics, and 1 million Korean patients with ankle sprains received Korean medicine treatment [10]. In addition to conventional treatments, complementary and alternative therapies such as acupuncture, herbs, cupping therapy, taping, and *chuna* have been used for the treatment of ankle sprains [11].

In Korean medicine, it is assumed that pain and swelling associated with ankle sprain are caused by blood stasis; therefore, activation of blood circulation is the main treatment principle for ankle sprain [12, 13]. Blood stasis is a significant pathology caused by disturbances in blood circulation [14, 15]. Blood stasis occurring within the body is termed blood stasis syndrome, which is characterized by symptoms such as pain in a fixed position, nyctalgia, dark-purple discoloration of the tongue and face, infraorbital darkness, sublingual varicosis, blood spots under the skin or tongue, and an astringent pulse [16]. This phenomenon is termed “EoHyeol” in Korean, “Yu Xue” in Chinese, and “Oketsu” in Japanese [17].

Dangguixu-san (DS), which is composed of *Angelicaegigantis Radix*, *PaeoniaeRadix*, *Linderae Radix*, *Cyperi Rhizoma*, *Sappan Lignum*, *Carthami Flos*, *Persicae Semen*, *Cinnamomi Cortex*, and *Glycyrrhizae Radix et Rhizoma*, promotes blood circulation and relieves blood stasis; therefore, it is most frequently used for the treatment of traumatic ecchymosis and pain [18, 19] and is recommended for the treatment of ankle sprain [11]. This formula is also known as Dangkwisoo-san in Korean and Tokishusan in Japanese [18, 20].

Although DS is used for the treatment of ankle sprain in Korean medicine, evidence regarding its efficacy is insufficient. Therefore, we performed a randomized controlled trial to investigate the efficacy and safety of DS for the treatment of acute lateral ankle sprain (ALAS).

## Methods

This study followed the Standard Protocol Items: Recommendations for Interventional Trials (SPIRIT) and Consolidated Standards of Reporting Trials (CONSORT) guidelines. The methods have been reported in detail in a previous study [21].

### Study design

This study was designed as a prospective, double-blind, multicenter, randomized, parallel-arm, placebo-controlled clinical trial with a 1:1 allocation ratio. The original study protocol [21] involved a single-center setting, which was changed to a multicenter setting for adequate participant enrolment and achievement of the target sample size. This change in the study design was approved by the Ministry of Food and Drug Safety and the Institutional Review Boards (IRBs) of Semyung University Korean Medicine Hospital in Chungju, and DongShin University Gwangju Korean Medicine Hospital. Forty-eight participants (28 from Semyung University Korean Medicine Hospital in Chungju and 20 from DongShin University Gwangju Korean Medicine Hospital) who met the inclusion criteria were randomly allocated to a DS (*n* = 24) or placebo (*n* = 24) group.

### Ethical considerations

This study was conducted in accordance with the Declaration of Helsinki, and version 0.9 of the protocol was approved by the Ministry of Food and Drug Safety (31244; approval date, April l4, 2017) and the IRB of Semyung University Korean Medicine Hospital (1705-07; approval date, May 8, 2017) before trial initiation. Version 1.0 was approved by the IRB of DongShin University Gwangju Korean Medicine Hospital (DSGOH-046; approval date, December 18, 2017). The trial was registered at the Clinical Research Information Service (cris.nih.go.kr; registration number, KCT0002374; registration date, July 11, 2017).

### Participant recruitment

Participants were recruited at Semyung University Korean Medicine Hospital in Chungju and DongShin University Gwangju Korean Medicine Hospital. We submitted our study protocol to CRIS on June8, 2017, and revised and resubmitted it on June 22, 2017. Considering the possibility of participant recruitment within the study period, we began recruitment on June30, 2017, before registration of the trial (July11, 2017).

### Participation

The inclusion criteria were as follows: aged > 19 years, occurrence of grade I or II ALAS within 72 h before enrolment, and voluntary provision of written informed consent. Grade I ankle sprain was diagnosed when there was no loss of function or ligament laxity (i.e., negative anterior drawer and talar tilt test results), little or no hemorrhage, no point tenderness, a decrease of ≤ 5° in the total ankle motion, and swelling measuring ≤ 0.5 cm. Patients with some loss of function, positive anterior drawer test findings (indicating anterior talofibular ligament involvement), negative talar tilt test findings (indicating no calcaneofibular ligament involvement), hemorrhage, point tenderness, a decrease of > 5° and < 10° in the total ankle motion, and swelling measuring > 0.5 cm and < 2.0 cm were diagnosed with grade II ALAS [22].

The exclusion criteria were as follows: fracture confirmed on X-ray or grade III ankle sprain (near total loss of function, positive anterior drawer and talar tilt test findings, hemorrhaging, extreme point tenderness, decrease of > 10° in the total ankle motion, and swelling measuring > 2.0 cm) [22]; a history of fracture in the same ankle during the previous year; serious disease conditions (e.g., cancer; kidney, liver, and central nervous system diseases; dementia; and blood clotting abnormalities such as hemophilia); motor or sensory disturbance due to nervous system disorders in the same leg; pregnancy or breastfeeding; use of drugs such as nonsteroidal anti-inflammatory drugs (NSAIDs), pain relievers, and steroids for pain relief from the time of trauma to participation in the clinical trial (excluding the use of adherent inflammatory pain relievers on the day of screening); impaired hepatic (alanine aminotransferase level, ≥ 80 IU/L) or renal (creatinine level, ≥ 2 mg/dL) function; ineligibility for participation because of a history of gastrointestinal diseases that could affect absorption of the trial medication, as judged by the principal investigator; genetic conditions such as galactose intolerance, Lapp lactase deficiency, and glucose–galactosemal absorption; a history of hypersensitivity to the components of the trial medication; and participation in other clinical trials within 4 weeks of screening for the present study or concurrent participation in other clinical trials.

The dropout criteria were as follows: < 80% compliance with the protocol procedures, incidence of serious adverse events (AEs), reluctance for continued participation in the trial, incomplete data that could influence the results, and large protocol error or significant deviation in implementation.

### Randomization and blinding

After the acquisition of written informed consent and completion of baseline measurements, the 48 enrolled participants were assigned serial numbers generated using a randomization tool (http://www.radomization.com) and randomly allocated to a DS (*n* = 24) or placebo (*n* = 24) group. The serial number codes were inserted into opaque envelopes that were sealed and stored in a double-locked cabinet. The participants, investigators, and outcome assessors remained blinded to the treatment allocation until study completion. A CRC generated the allocation sequence, enrolled the participants, and assigned them to the groups.

### Intervention

Considering that DS is mainly used with acupuncture in the clinical situation, this study used acupuncture as the basic treatment. Both groups received acupuncture treatment once a day for 5 consecutive days and the trial medication (DS/placebo capsule) 3 times a day for 7 consecutive days.

For the acupuncture treatment, only sterile, stainless, disposable acupuncture needles (size 0.25 × 30 mm; Dong Bang Acupuncture, Inc., Boryeong, Republic of Korea; product no. A84010.02) with guide tubes were vertically inserted into the ST36, ST41, BL60, BL62, KI3, KI6, GB39, and GB40 acupuncture points on the affected side [11]. The depth of insertion was 10 to 20 mm, depending on the location of the needle [23]. After insertion, the needles were maintained in position for 15 min during each session. Manual or electrical stimulation was not applied.

The trial medications included DS and placebo capsules, which were identical in appearance, taste, and smell. The DS formulation was composed of granular extracts of nine herbal substances and provided as a gray–brown powder contained in a 0.6-g pink capsule. The placebo was composed of 300 mg of corn starch, 150 mg of lactose hydrate, 1.5 mg of magnesium stearate, and 48.5 mg of caramel colorant. Both medications were administered at a dosage of 3 capsules 3 times daily (total daily dosage, nine capsules) for a period of 1 week. The manufacturing process for the DS capsules was as follows. First, 0.63 g of *Angelicaegigantis Radix*, 0.42 g of *Paeoniae Radix*, 0.42 g of *Linderae Radix*, 0.42 g of *Cyperi Rhizoma*, 0.42 g of *Sappan Lignum*, 0.33 g of *Carthami Flos,*0.29 g of *Persicae Semen*, 0.25 g of *Cinnamomi Cortex*, and 0.21 g of *Glycyrrhizae Radix et Rhizoma* were added to the extractor. The mixture was extracted with 8–10 volumes of purified water at 80–100 °C for 3–4 h. The extract was filtered (100 mesh), concentrated under reduced pressure at ≤ 60 °C, and dried to obtain 350 mg of a granular extract. Then, 45 mg of lactose hydrate, 100 mg of corn starch, and 5 mg of magnesium stearate were mixed into the granular extract, and the mixture was converted into granules, dried, crushed, and filled into capsules.

Capsules were sourced from Kyungjin Pharmaceutical Co., Ltd. (Icheon, Gyeonggi-do, Republic of Korea; product no. 02190). The medication was dispensed as 0.6-g capsules packed in sealed boxes containing a 1-week dose.

Between the preintervention period and a 4-week period after treatment completion, participants were restricted from consuming the following drugs: NSAIDS, pain relievers, steroids, or adherent inflammatory pain relievers; drugs containing potassium, licorice, glycyrrhizic acid, furosemide, ethacrynic acid, or trichloromethazine; and drugs containing any of the main ingredients of DS. Participants were asked to return their medication boxes to allow enumeration of leftover capsules and for compliance assessment.

### Outcome measurements

The primary outcome was the change in the visual analog scale (VAS) score for pain, and the secondary outcomes were changes in Foot and Ankle Outcome Scores (FAOS), edema, European Quality of Life Five-Dimension-Five-Level Scale (EQ-5D-5L) scores, and the number of recurrent ankle sprains. VAS, FAOS, and edema measurements were performed at baseline (week 0; before intervention), 5 days after the first intervention (week1; the end of the intervention), and 4 weeks after treatment completion (week 5). EQ-5D-5L measurements were conducted at weeks 0, 1, and 5 and additionally at 26 weeks after treatment completion (week 27). The number of recurrent ankle sprains was assessed at 4 (week 5), 8 (week 9),12 (week 13), and 26 weeks (week 27) after treatment completion.

VAS is a validated, subjective measure for acute and chronic pain. Scores are recorded by manual marking of a point on a 10-cm line representing a continuum between “no pain” and “worst pain imaginable” [24]. Participants were asked to mark a point representing the severity of their pain. Scores were recorded in millimeters, and the total score ranged from 0 to 100 mm [25].

FAOS is a self-administered questionnaire specific for foot and ankle injuries that was developed to assess weekly changes in symptoms and functions after these injuries. It includes 42 items assessing five domains of recovery: pain (nine items), other symptoms (seven items), activities of daily living (17 items), sports and recreational activities (five items), and foot and ankle-related quality of life (four items) [26].

Edema was measured in centimeters via the figure-of-eight method. The measuring tape was applied across the following landmarks in a figure-of-eight fashion as follows: navicular tuberosity, distal tip of the lateral malleolus, distal tip of the medial malleolus, and base of the fifth metatarsal. The resulting value was compared with the corresponding value for the healthy ankle [27].

The EQ-5D is a generic instrument for assessing health-related quality of life. It classifies health into five dimensions: mobility, self-care, usual activities, pain/discomfort, and anxiety/depression. Each dimension has three response categories: none, some, and extreme/unable to. The EQ-5D-5L is a new version of EQ-5D that includes five levels of severity (none, slight, moderate, severe, extreme/unable to) for each of the existing five EQ-5D dimensions [28, 29].

Ankle sprain recurrence was defined as the repeated occurrence of ankle sprain as a result of sports participation or other daily activities, with one or more of the following consequences: (1) termination of the sports activity, limited participation in the next planned sports activity, inability to go to work/school the following day, and the need for medical attention (ranging from onsite care administered by a general practitioner to personal care administered by a sports physician) [30].

### Sample size calculation

Unfortunately, we did not have a preliminary study for reference, and previous similar studies are limited. Considering our limited funds and the maximum number of participants available at the two centers within the study period, our maximum possible sample size was 48. Accordingly, we adopted a pilot study design with 24 participants in each group.

As our study was a pilot study, the sample size was not sufficient for determining the efficacy of DS for ALAS. Our study will provide preliminary evidence for the efficacy and safety of DS for ALAS.

### Statistical analyses

Under IRB approval, we revised the statistical analysis procedure documented in the original study protocol. We performed per-protocol (PP) analyses for the assessment of efficacy and analyzed a supplementary full analysis set (FAS). Missing values were implemented by the last observation carried forward method. We compared the results of PP analyses and analyses of the FAS. All analyses were performed by blinded biostatisticians using SPSS version 20.0 software (IBM Corp, Armonk, NY, USA) and two-sided significance tests with a 5% significance level. Continuous variables are presented as means and standard deviations (SDs), and categorical variables are presented as count frequencies and percentages.

Baseline data were collected and compared using independent *t* tests, *χ*^2^ tests, and Fisher’s exact tests. Within-group differences in the outcome measurements (week 0 vs. week 1, week 0 vs. week 5, week 1 vs. week 5) were evaluated using the Wilcoxon signed-rank test (nonparametric test) and repeated measures ANOVA (Friedman tests). Values of VAS, edema, EQ-5D-5L, and FAOS were compared by repeated-measures ANOVA across two to three time points (week 0, week 1, week 5). Changes in the outcome measurements (week 0 to week 1, week 0 to week 5, week 1 to week 5) were compared between groups using the Mann–Whitney *U* test (nonparametric test). The number of recurrence ankle sprains at the different time points (week 5, week 9, week 13, week 27) were compared between groups using the Mann–Whitney *U* test (nonparametric test). For sub-analysis, participants were divided into grade I and grade II groups according to the severity of the ankle sprain, and differences in VAS, edema, EQ-5D-5L, and FAOS changes (week0 to week1, week0 to week5, and week1 to week5) between the two groups were evaluated using the Mann–Whitney U-test (nonparametric test).

## Results

### Participants

**Fig. 1:**
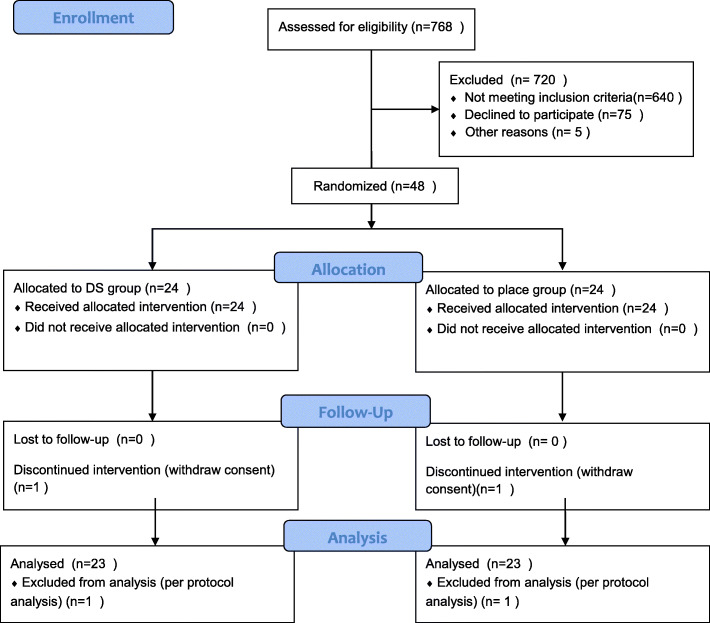
CONSORT 2010 flow diagram for the trial

We recruited participants between June 30, 2017, and December 24, 2018, at Semyung University Korean Medicine Hospital in Chungju and between January 17, 2018, and February 15, 2019, at DongShin University Gwangju Korean Medicine Hospital. During the study period, 768 patients were assessed for eligibility and 720 were excluded. Eventually, 48 patients were included in this study and randomly assigned to a DS group (*n* = 24) and a placebo group (*n* = 24). One patient did not complete the treatment in each group. The results of PP analysis for the assessment of efficacy were not different from those for the FAS. Thus, data for 46 patients with ankle sprain (DS group, *n* = 23 [grade I, *n* = 12; II, *n* = 11]; placebo group, *n* = 23 [grade I, *n* = 11; II, *n* = 12]) were included in the final analysis (Fig. 1).

### Baseline characteristics

The baseline demographic characteristics and study variables for the 46 patients in the two groups are presented in Table 1. There were no significant differences in any parameter between the two groups (*P* > 0.05; Table 1).

**Table 1:** Homogeneity tests for baseline demographic characteristics and study variables for 46 patients with acute lateral ankle sprain

Dependent variables	DS (*n* = 23)	Placebo (*n* = 23)	*p* or *x*^2^ (*P*)
	Mean (SD) or *n* (%)	Mean (SD) or *n* (%)	
Age (years)	43.57 (14.99)	43.74 (15.42)	− 0.52 (.605)^a^
Gender (female)	14 (60.9%)	10 (43.5%)	1.39 (.188)^b^
Lesion side (Lt)	13 (56.5%)	9 (39.1%)	1.39 (.188)^b^
Sprains injury rating (grade 1)	12 (52.2%)	11 (47.8%)	0.09 (1.00)^b^
Duration	1.48 (0.73)	1.57 (0.51)	− 0.65 (.514)^b^
BMI	25.44 (3.49)	23.81 (2.44)	− 1.78 (.075)^a^
VAS of pain	0.67 ± 0.89	0.52 ± 0.67	− 0.44 (.658) ^a^
Degree of edema	44.87 ± 17.02	44.04 ± 16.49	− 0.16 (.876)^a^
EQ-5D-5L	10.48 ± 3.06	10.17 ± 2.60	− 0.17 (.868)^a^
FAOS	307.43 ± 82.19	299.54 ± 76.45	− 0.60 (.546) ^a^
FAOS Symptom/Rigidity	67.10 ± 18.05	66.51 ± 13.46	− 0.49 (.628)^a^
FAOS ache	60.60 ± 17.38	60.60 ± 17.38	− 0.06 (.955) ^a^
FAOS Function, everyday life	74.41 ± 17.87	72.04 ± 15.84	− 0.91 (.362)^a^
FAOS Features, Sports/Leisure	49.67 ± 24.78	43.93 ± 24.30	− 0.74 (.460)^a^
FAOS Quality of life	58.30 ± 19.93	54.92 ± 22.45	− 0.49 (.627)^a^

^a^*t* test; ^b^*x*^2^ test

### Primary and secondary outcomes

After 1 week of treatment, we observed significant improvements in VAS-pain scores, the degree of edema, EQ-5D-5L scores, total FAOS, FAOS Symptom/Rigidity, FAOS Ache, FAOS Function Everyday Life, FAOS Features Sports/Leisure, and FAOS Quality of Life (Table 2) in both groups.

**Table 2:** Changes in outcome measures (week 0 vs. week 1, week 0 vs. week 5) after treatment completion in patients who received Dangguixu-san or placebo for acute lateral ankle sprain (*n* = 23 each)

Groups	Dependent variables	Week 0 (M ± SD)	Week 1 (M ± SD)	Week 5 (M ± SD)	Difference (w1–w0)	*Z* (*P*)^a^	Difference (w5–w0)	*Z* (*P*)^a^	*x*^2^ (*P*)^b^
DS group (*n* = 23)	Degree of edema	0.67 ± 0.89	0.23 ± 0.43	0.21 ± 0.33	− 0.44 ± 0.93	− 2.57 (0.010)	− 0.45 ± 0.83	− 2.47 (0.014)	6.48 (0.039)
	VAS of pain	44.87 ± 17.02	24.96 ± 18.27	12.74 ± 16.20	− 19.91 ± 21.62	− 3.09 (0.002)	− 32.13 ± 23.65	−3.75 (< 0.001)	32.89 (< 0.001)
	TOTAL EQ-5D-5L	10.48 ± 3.06	8.35 ± 2.85	6.70 ± 2.77	−2.13 ± 2.69	−3.04 (0.002)	−3.78 ± 4.22	− 3.49 (< 0.001)	26.42 (< 0.001)
	TOTAL FAOS	307.43 ± 82.19	368.55 ± 74.72	430.09 ± 79.82	61.13 ± 72.52	−3.13 (0.002)	122.66 ±107.34	−3.65 (< 0.001)	24.02 (< 0.001)
Placebo group (*n* = 23)	Degree of edema	0.52 ± 0.67	0.27 ± 0.54	0.12 ± 0.44	−0.24 ± 0.53	−2.11 (0.035)	−0.40 ± 0.70	−2.60 (0.009)	8.03 (0.018)
	VAS of pain	44.04 ± 16.49	19.26 ± 14.14	14.87 ± 12.82	−24.78 ± 12.83	−4.07(< 0.001)	− 29.17 ± 16.26	−3.94 (< 0.001)	36.56 (< 0.001)
	TOTAL EQ-5D-5L	10.17 ± 2.61	7.61 ± 1.83	6.65 ± 1.70	−2.57 ± 1.78	−3.94(< 0.001)	− 3.52 ± 2.84	− 3.72 (< 0.001)	32.84 (< 0.001)
	TOTAL FAOS	299.54 ± 76.45	383.45 ± 66.80	428.00 ± 59.18	83.91 ± 74.37	−3.68(< 0.001)	128.46 ± 85.78	−3.83 (< 0.001)	28.17 (< 0.001)

^a^Wilcoxon signed-rank test; ^b^Repeated measures ANOVA (Friedman test)

Repeated-measures ANOVA showed no significant interaction between time and group with respect to all study variables (Table 3).

**Table 3:** Results of repeated-measures ANOVA for the outcomes of treatment in patients who received Dangguixu-san or placebo for acute lateral ankle sprain (*n* = 23 each)

Dependent variables	Group (*n*)	Source	SS	df	Mean square	*F*	*p*
VAS of pain	DS (*n* = 23)	Time	23,121.80	2	11,560.90	67.42	< 0.001
	Placebo (*n* = 23)	Group* Time	351.19	2	179.59	1.05	0.355
Degree of edema	DS (*n* = 23)	Time	4.65	2	2.32	9.17	< 0.001
	Placebo (*n* = 23)	Group* Time	0.23	2	0.12	0.46	0.633
Total EQ-5D-5L	DS (*n* = 23)	Time	315.13	2	157.57	32.65	< 0.001
	Placebo (*n* = 23)	Group* Time	2.84	2	1.42	0.29	0.746
Total FAOS	DS (*n* = 23)	Time	365,514.69	2	172,757.35	51.71	< 0.001
	Placebo (*n* = 23)	Group* Time	3221.20	2	1612.10	0.46	0.635

* interaction between time and group

**Fig. 2:**
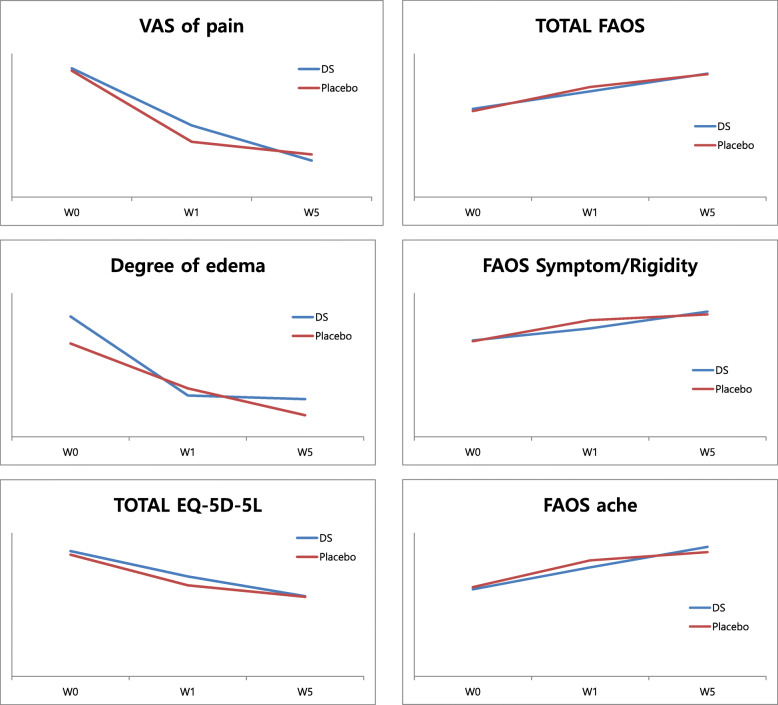
Comparison of changes in outcome measurements between DS and placebo group

Changes in VAS-pain scores, FAOS Symptom/Rigidity, and FAOS Ache from week 1 to week 5 were significantly different between the two groups (Table 4, Fig. 2), with superior outcomes for DS treatment.

**Table 4:** Comparison of changes in outcome measurements between patients who received Dangguixu-san and those who received placebo for acute lateral ankle sprain (*n* = 23 each)

Dependent variables	Group (*n*)	Difference (w1–w0)	*Z* (*p*)^a^	Difference (w5–w0)	*Z* (*p*)^a^	Difference (w5–w1)	*Z* (*p*)^a^
VAS of pain	DS (*n* = 23)	19.91 ± 21.62	− 0.82 (0.412)	32.13 ± 23.65	− 0.63 (0.527)	12.22 ± 22.08	− 2.59 (0.010)
	Placebo (*n* = 23)	24.78 ± 12.83		29.17 ± 16.26		4.39 ± 10.71	
Degree of edema	DS (*n* = 23)	0.44 ± 0.93	− 0.66 (0.512)	0.45 ± 0.83	− 0.09 (0.929)	0.01 ± 0.59	− 0.96 (0.338)
	Placebo (*n* = 23)	0.24 ± 0.53		0.40 ± 0.70		0.15 ± 0.61	
Total EQ-5D-5L	DS (*n* = 23)	2.13 ± 2.69	− 0.51 (0.609)	3.78 ± 4.22	− 0.48 (0.633)	1.65 ± 4.13	− 1.29 (0.198)
	Placebo (*n* = 23)	2.57 ± 1.78		3.52 ± 2.84		0.96 ± 2.14	
Total FAOS	DS (*n* = 23)	61.13 ± 72.52	− 0.91 (0.362)	122.66 ± 107.34	− 0.41 (0.684)	61.53 ± 99.49	− 1.15 (0.249)
	Placebo (*n* = 23)	83.91 ± 74.37		128.46 ± 85.78		44.55 ± 53.31	
FAOS Symptom/Rigidity	DS (*n* = 23)	8.36 ± 15.97	− 1.42 (0.155)	20.16 ± 21.87	− 0.08 (0.939)	11.80 ± 17.34	− 2.54 (0.011)
	Placebo (*n* = 23)	14.69 ± 12.83		18.74 ± 17.07		4.05 ± 10.47	
FAOS ache	DS (*n* = 23)	15.52 ± 14.41	− 0.80 (0.426)	29.29 ± 22.83	− 1.01 (0.312)	14.25 ± 20.91	− 2.69 (0.007)
	Placebo (*n* = 23)	18.60 ± 16.60		24.41 ± 18.33		5.81 ± 10.12	

^a^Mann-Whitney *U* test

According to the results of sub-analysis based on symptom severity, there were no significant differences in all variables between the two groups of patients with grade I ALAS (Table 5). However, changes in FAOS Ache from week 0 to week 5 and changes in VAS scores, total FAOS, and EQ-5D-5L scores from week 1 to week 5were significantly different between the two groups of patients with grade II ALAS (Table 6), with superior outcomes for DS treatment.

**Table 5:** Comparison of changes in outcome measurements between patients who received Dangguixu-san and those who received placebo for grade I acute lateral ankle sprain (*n* = 23)

Dependent variables	Group (*n*)	Week 0 (M ± SD)	Week 1 (M ± SD)	Week 5 (M ± SD)	Difference (w1–w0)	*Z* (*p*)^a^	Difference (w5–w0)	*Z* (*p*)^a^	Difference (w5–w1)	*Z* (*p*)^a^
VAS of pain	DS (*n* = 12)	39.33 ± 14.51	19.50 ± 11.70	16.67 ± 21.46	− 19.83 ± 15.02	− 0.31 (0.755)	− 22.67 ± 24.64	− 0.56 (0.577)	− 2.83 ± 20.66	− 0.32 (0.750)
	Placebo (*n* = 11)	43.91 ± 21.19	22.82 ± 12.24	14.73 ± 10.56	− 21.09 ± 15.00		−29.18 ± 19.81		−8.09 ± 8.62	
Degree of edema	DS (*n* = 12)	0.17 ± 0.39	0.17 ± 0.58	0.25 ± 0.45	0.03 ± 0.36	− 0.24 (0.812)	0.03 ± 0.45	− 0.79 (0.431)	0.00 ± 0.67	− 0.69 (0.488)
	Placebo (*n* = 11)	0.09 ± 0.54	0.09 ± 0.30	0.00 ± 0.00	− 0.06 ± 0.24		−0.08 ± 0.25		−0.02 ± 0.26	
TOTAL EQ-5D-5L	DS (*n* = 12)	9.33 ± 1.78	7.08 ± 1.56	7.50 ± 3.66	− 2.25 ± 1.82	− 0.84 (0.399)	− 1.83 ± 3.90	− 0.77 (0.439)	0.42 ± 4.10	− 0.51 (0.609)
	Placebo (*n* = 11)	10.73 ± 2.90	7.73 ± 1.49	7.18 ± 1.89	− 3.00 ± 2.10		− 3.55 ± 3.83		− 0.55 ± 2.62	
TOTAL FAOS	DS (*n* = 12)	349.28 ± 60.85	393.40 ± 51.89	403.23 ± 98.09	44.13 ± 52.35	− 0.19 (0.854)	53.95 ± 86.99	− 1.05 (0.295)	9.83 ± 100.83	− 0.86 (0.389)
	placebo (*n* = 11)	314.15 ± 88.63	376.45 ± 62.75	410.71 ± 63.41	62.31 ± 84.87		96.56 ± 106.41		34.25 ± 60.46	

^a^Mann-Whitney *U* test

**Table 6:** Comparison of outcome measurements between patients who received Dangguixu-san and those who received placebo for grade II acute lateral ankle sprain(n = 23)

Dependent variables	Group (*n*)	Week 0 (M ± SD)	Week 1 (M ± SD)	Week 5 (M ± SD)	Difference (w1–w0)	*Z* (*p*)^a^	Difference (w5–w0)	*Z* (*p*)^a^	Difference (w5–w1)	*Z* (*p*)^a^
VAS of pain	DS (*n* = 11)	50.91 ± 18.14	30.91 ± 22.56	8.45 ± 5.65	−20.00 ± 27.93	− 0.71 (0.479)	− 42.45 ± 18.38	− 1.54 (0.123)	− 22.45 ± 19.54	− 3.03 (0.002)
	Placebo (*n* = 12)	44.17 ± 11.65	16.00 ± 15.47	15.00 ± 15.08	− 28.17 ± 9.93		−29.17 ± 13.11		−1.00 ± 11.65	
Degree of edema	DS (*n* = 11)	1.36 ± 1.03	0.27 ± 0.47	0.36 ± 0.50	− 0.95 ± 1.10	− 1.31 (0.189)	− 0.98 ± 0.83	− 0.85 (0.396)	0.03 ± 0.51	− 0.72 (0.473)
	Placebo (*n* = 12)	1.08 ± 0.79	0.50 ± 0.67	0.25 ± 0.62	− 0.41 ± 0.67		− 0.68 ± 0.86		−0.28 ± 0.81	
TOTAL EQ-5D-5L	DS (*n* = 11)	11.73 ± 3.72	9.73 ± 3.35	5.82 ± 0.75	− 2.00 ± 3.49	− 0.88 (0.880)	− 5.91 ± 3.59	− 1.85 (0.065)	− 3.91 ± 2.88	− 2.66 (0.008)
	Placebo (*n* = 12)	9.67 ± 2.31	7.50 ± 2.15	6.17 ± 1.40	− 2.17 ± 1.40		− 3.50 ± 1.68		−1.33 ± 1.61	
TOTAL FAOS	DS (*n* = 11)	261.77 ± 79.97	341.45 ± 88.16	459.39 ± 40.28	79.67 ± 88.48	− 0.31 (0.758)	197.62 ± 71.97	− 1.23 (0.218)	117.95 ± 62.34	− 2.59 (0.010)
	Placebo (*n* = 12)	286.15 ± 64.35	389.86 ± 72.46	443.85 ± 52.68	103.71 ± 60.16		157.70 ± 49.73		53.99 ± 46.46	
FAOS ache	DS (*n* = 11)	52.00 ± 15.63	69.69 ± 14.41	95.19 ± 5.28	17.68 ± 14.67	− 1.05 (0.294)	43.18 ± 14.36	− 2.35 (0.019)	25.50 ± 11.79	− 3.49 (< 0.001)
	Placebo (*n* = 12)	59.67 ± 11.50	82.63 ± 16.29	89.35 ± 10.51	22.92 ± 14.08		29.64 ± 9.29		6.73 ± 8.16	

^a^Mann-Whitney *U* test

The DS and placebo groups showed no significant differences in the number of recurrent ankle sprains at weeks 5, 9, 13, and 27 and changes in EQ-5D-5L scores from week 0 to week 27 (Tables 7 and 8).

**Table 7:** Number of recurrences at different time points in patients who received Dangguixu-san or placebo for acute lateral ankle sprain (*n* = 23 each)

Dependent variables	DS (*n* = 23)	Placebo (*n* = 23)	*z* (*p*)^a^
	(M ± SD)	(M ± SD)	
Total relapse	0.61 ± 1.34	0.13 ± 0.46	− 1.83 (0.068)
**Week 5 relapse**	0.13 ± 0.34	0.09 ± 0.42	− 0.96 (0.334)
**Week 9 relapse**	0.00 ± 0.00	0.00 ± 0.00	− 0.00 (1.00)
**Week 13 relapse**	0.26 ± 1.05	0.04 ± 0.21	− 0.62 (0.538)
**Week 27 relapse**	0.22 ± 0.52	0.00 ± 0.00	− 2.61 (0.039)

^a^Mann-Whitney *U* test

**Table 8:** Comparison of changes in EQ-5D-5L scores from baseline to 26 weeks after treatment between patients who received Dangguixu-san and those who received placebo for acute lateral ankle sprain (*n* = 23 each)

Dependent variables	Group(n)	Week 0 (M ± SD)	Week 27 (M ± SD)	Difference (w27–w0)	*Z* (*p*)^a^
Total EQ-5D-5L	DS (*n* = 23)	10.48 ± 3.06	5.48 ± 1.04	− 5.00 ± 3.02	− 0.50 (0.618)
	Placebo (*n* = 23)	10.17 ± 2.61	5.61 ± 1.27	− 4.57 ± 2.98	

^a^Mann-Whitney *U* test

### Safety evaluation

For safety evaluation, the incidence of AEs and the results of clinical laboratory tests performed before (week 0) and after (week 1) treatment were compared between the two groups. AEs were recorded on a case report form after evaluation of their relationships with the intervention. No intervention-related AEs occurred in the DS and placebo groups. There were no significant treatment-induced changes in all clinical laboratory parameters except alaninetransaminase and creatinine, which exhibited significant increases in the placebo group relative to the changes in the DS group. However, the increases were within the normal ranges for both parameters (Table 9).

**Table 9:** Comparison of change in clinical laboratory parameters between patients who received Dangguixu-san and those who received placebo for acute lateral ankle sprain (*n* = 23 each)

Dependent variables			Week 0 (M ± SD)	Week 1 (M ± SD)	Difference (w1–w0)	*Z*	*p*
Biochemistry	ALT (SGPT)	DS (*n* = 23)	24.65 ± 11.58	23.00 ± 10.44	− 1.65 ± 4.75	− 1.99	0.047
		Placebo (*n* = 23)	20.61 ± 10.92	21.96 ± 11.93	1.35 ± 4.25		
	Cr	DS (*n* = 23)	0.82 ± 0.21	0.80 ± 0.21	−0.02 ± 0.15	− 2.05	0.040
		Placebo (*n* = 23)	0.78 ± 0.17	0.84 ± 0.19	0.07 ± 0.14		

## Discussion

To our knowledge, this is the first randomized, double-blind, placebo-controlled study investigating the effects of DS with regard to the reduction of pain and edema; recovery of function, activities of daily living, and quality of life; and recurrence in patients with ALAS.

Because studies of herbal medicines for ankle sprain are few, our study design (i.e., the treatment and evaluation schedules) was based on the designs of several acupuncture studies for ankle sprain [31]. Considering that DS is primarily used with acupuncture in clinical situations, we used acupuncture treatment as the basic treatment in the present study.

Biological ligament healing can be divided into three phases: inflammatory phase, proliferation phase, and remodeling or maturation phase [32]. The present study included patients with ankle sprain in the inflammatory phase (occurrence within 72 h before enrolment). There is good evidence for the usefulness of immobilization and occasionally, surgical correction for the management of grade III ankle sprains [33]. Therefore, we excluded patients with grade III ankle sprain. We also excluded patients who were using analgesics that could affect the study outcomes and those at risk of AEs associated with DS treatment.

There were several major findings in the present study. First, relative to placebo treatment, DS did not induce evident beneficial changes from baseline (week 0) to the end of treatment (week 1) in patients with grade I or II ALAS; however, it resulted in significant pain reduction and symptom alleviation over 4 weeks after treatment completion (week 1 to week 5). Second, in patients with grade II ALAS, DS resulted in significant pain reduction over 5 weeks after the start of treatment (week 0 to week 5) as well as significant pain reduction and recovery of function, activities of daily living, and quality of life over 4 weeks after treatment completion (week 1 to week 5). Third, DS did not induce AEs or significant negative changes in clinical laboratory parameters during or after treatment.

Both DS and placebo resulted in significant pain and edema reduction and recovery of function, activities of daily living, and quality of life in patients with ALAS. However, there were no significant positive effects of DS immediately after treatment completion. These results may be related to the therapeutic effects of the intervention, which was the basic treatment in both groups in the present study.

When patients with grade I or grade II ALAS were separately analyzed, it was found that DS induced significant therapeutic effects in patients with grade II ALAS, with pain reduction starting from week 0 and functional recovery starting from week 1. These results may be related to the characteristics of grade I and II ALAS and the effects of DS on blood stasis associated with ankle sprain. Ankle ligament sprains are generally graded on the basis of severity. Grade I sprains are characterized by mild stretching of the ligaments without macroscopic rupture or joint instability, while grade II (moderate) sprains involve partial rupture of the ligament with moderate pain and swelling. There are functional limitations and slight to moderate instability. Typically, patients present with problems in weight bearing [22]. The distinction between grade I or grade II/III injuries is important from the perspective of both treatment and prognosis. It is suggested that a grade I sprain does not need any treatment because functional instability or recurrent sprain is less frequent after ligament sprains than after ligament ruptures [34].

Increased interleukin-6 (IL-6) levels are clinically correlated with exacerbation of trauma and the associated inflammation; therefore, IL-6 is an important biomarker of trauma [35, 36]. DS, which has been conventionally prescribed for the treatment of inflammation caused by physical trauma, has been found to lower the IL-6 level. In addition, it was found to activate Nrf2, a key anti-inflammatory factor, and suppress nuclear factor-kappa beta, a well-established pro-inflammatory factor, in vitro [37]. Hematoma and tenderness associated with grade II ankle sprains are similar to the characteristic symptoms of blood stasis, such as blood spots under the skin and pain in a fixed position. Thus, DS, which promotes blood circulation and relieve blood stasis, resulted in significant long-term effects in patients with grade II ALAS in the present study.

This study has some limitations. First, it was a pilot study with a small sample size. Therefore, the number of subjects included in the final analysis was small. Second, we adopted acupuncture as a basic treatment, which might have led to biased results. In our opinion, it would be better to adopt other conventional treatments (e.g., usual care, physiotherapy) for ankle sprain or DS treatment alone. Third, we included patients with grade I or grade II ALAS as participants, which might have led to biased results. We believe that future studies should include only grade II ALAS, which exhibits features similar to the characteristic symptoms of blood stasis. Fourth, our findings were limited to acute grade I or grade II ALAS. Further studies could analyze the effects of DS in patients with chronic or grade III ALAS.

## Conclusions

In conclusion, our results have established clinical evidence that DS combined with acupuncture did not show beneficial effects compared to placebo combined with acupuncture from baseline to the end of treatment. However, relative to placebo combined with acupuncture, DS combined with acupuncture was a safe treatment with positive long-term effects in terms of pain reduction and symptom alleviation in patients with grade I or II ALAS. Moreover, it was found to reduce pain and gradually restore function, activities of daily living, and quality of life in patients with grade II ALAS. This suggests that DS combined with acupuncture may be a potential treatment option along with conventional treatment for ALAS.

## Supplementary Information

**Additional file 1.**

## Data Availability

The datasets used and/or analyzed during the current study are available from the corresponding author on reasonable request.

## Abbreviations

AEs: Adverse events
ALAS: Acute lateral ankle sprain
ANOVA: Analysis of variance
CAI: Chronic ankle instability
CONSORT: Consolidated Standards of Reporting Trials
CRC: Clinical research coordinator
DS: Dangguixu-san
EQ-5D-5L: European Quality of Life-Five Dimension-Five Level Scale
FAS: Full analysis set
FAOS: Foot and Ankle Outcome Score
IRB: Institutional Review Board
NSAID: Nonsteroidal anti-inflammatory drug
PP: Per-protocol
SPIRIT: Standard Protocol Items of the Recommendations for Interventional Trials
VAS: Visual analog scale
