# Supplementary material for: Effects of Dangguixu-san in patients with acute lateral ankle sprain: a randomized controlled trial
Source: Trials. 2021 Mar 4;22:184. doi: 10.1186/s13063-021-05135-6 (PMC7934479; doi:10.1186/s13063-021-05135-6)
Supplement: Supplementary file 1 — Additional file 1. [file 13063_2021_5135_MOESM1_ESM.docx]

Supplementary Table S 1. **Changes in outcome measures (week0 vs. week1, week0 vs. week5) after treatment completion in patients who received Dangguixu-san or placebo for acute lateral ankle sprain (n=23 each)**

| Groups | Dependent  Variables | Week 0  (M±SD) | Week 1  (M±SD) | Week 5  (M±SD) | Difference  (w1-w0) | Z *(P)* * | Difference  (w5-w0) | Z *(P)* * | x² *(P)* ǂ |
| --- | --- | --- | --- | --- | --- | --- | --- | --- | --- |
| DS group  (n=23) | Degree of edema | 0.67 ±0.89 | 0.23 ±0.43 | 0.21 ±0.33 | -0.44±0.93 | -2.57 (0.010) | -0.45±0.83 | -2.47 (0.014) | 6.48  (0.039) |
|  | VAS of pain | 44.87 ±17.02 | 24.96 ±18.27 | 12.74 ±16.20 | -19.91±21.62 | -3.09 (0.002) | -32.13±23.65 | -3.75 (<0.001) | 32.89  (<0.001) |
|  | TOTAL  EQ-5D-5L | 10.48 ±3.06 | 8.35 ±2.85 | 6.70 ±2.77 | -2.13±2.69 | -3.04 (0.002) | -3.78±4.22 | -3.49 (<0.001) | 26.42  (<0.001) |
|  | TOTAL FAOS | 307.43 ±82.19 | 368.55 ±74.72 | 430.09±79.82 | 61.13 ±72.52 | -3.13 (0.002) | 122.66  ±107.34 | -3.65 (<0.001) | 24.02  (<0.001) |
|  | FAOS  Symptom/Rigidity | 67.10 ±18.05 | 75.45 ±12.39 | 87.25 ±16.27 | 8.36 ±15.97 | -2.17 (0.030) | 20.16±21.87 | -3.38 (0.001) | 15.56  (<0.001) |
|  | FAOS  ache | 60.62 ±16.98 | 75.95 ±13.88 | 90.20 ±14.00 | 15.52 ±14.41 | -3.58(<0.001) | 29.29±22.83 | -3.56 (<0.001) | 23.23  (<0.001) |
|  | FAOS  Function, everyday life | 74.41 ±17.87 | 84.71 ±15.68 | 92.45 ±14.72 | 10.30 ±19.60 | -2.63 (0.009) | 18.04±24.12 | -3.50 (<0.001) | 20.02  (<0.001) |
|  | FAOS  Features,  Sports/Leisure | 49.67 ±24.78 | 63.70 ±26.21 | 78.48 ±22.84 | 14.02 ±24.53 | -2.17 (0.030) | 28.80±30.07 | -3.46 (0.001) | 16.47  (<0.001) |
|  | FAOS  Quality of life | 58.30 ±19.93 | 68.77 ±17.87 | 81.71 ±19.21 | 10.47 ±21.06 | -2.38 (0.018) | 23.41±22.95 | -3.46 (0.001) | 21.40  (<0.001) |
| Placebo  group  (n=23) | Degree of edema | 0.52 ±0.67 | 0.27 ±0.54 | 0.12 ±0.44 | -0.24±0.53 | -2.11 (0.035) | -0.40±0.70 | -2.60 (0.009) | 8.03  (0.018) |
|  | VAS of pain | 44.04 ±16.49 | 19.26 ±14.14 | 14.87 ±12.82 | -24.78±12.83 | -4.07(<0.001) | -29.17±16.26 | -3.94 (<0.001) | 36.56  (<0.001) |
|  | TOTAL  EQ-5D-5L | 10.17 ±2.61 | 7.61 ±1.83 | 6.65 ±1.70 | -2.57±1.78 | -3.94(<0.001) | -3.52±2.84 | -3.72 (<0.001) | 32.84  (<0.001) |
|  | TOTAL FAOS | 299.54 ±76.45 | 383.45 ±66.80 | 428.00±59.18 | 83.91±74.37 | -3.68(<0.001) | 128.46±85.78 | -3.83 (<0.001) | 28.17  (<0.001) |
|  | FAOS  Symptom/Rigidity | 66.50 ±13.45 | 81.20 ±10.99 | 85.24 ±14.02 | 14.69±12.83 | -3.59(<0.001) | 18.74±17.07 | -3.31 (0.001) | 26.00  (<0.001) |
|  | FAOS  Ache | 62.18 ±15.56 | 80.78 ±13.25 | 86.59 ±12.72 | 18.60±16.60 | -3.64(<0.001) | 24.41±18.33 | -3.64 (<0.001) | 28.11  (<0.001) |
|  | FAOS  Function, everyday life | 72.04 ±15.84 | 88.09 ±10.11 | 94.04 ±7.60 | 16.05±14.60 | -3.67(<0.001) | 22.00±15.22 | -4.02 (<0.001) | 25.37  (<0.001) |
|  | FAOS  Features,  Sports/Leisure | 43.93 ±24.30 | 65.43 ±22.25 | 80.87 ±15.86 | 21.51±22.78 | -3.45 (0.001) | 36.94±24.98 | -3.86 (<0.001) | 29.59  (<0.001) |
|  | FAOS  Quality of life | 54.92 ±22.45 | 67.95 ±21.68 | 81.26 ±19.40 | 13.03±22.13 | -2.47 (0.014) | 26.34±22.22 | -3.67 (<0.001) | 23.17  (<0.001) |

**Supplementary Table S 2.** **Comparison of changes in outcome measurements between patients who received Dangguixu-san and those who received placebo for acute lateral ankle sprain (n=23 each)**

| Dependent Variables | Group(n) | Week 0  (M±SD) | Difference  (w1-w0) | *Z(p)*^*^ | Difference  (w5-w0) | *Z(p)*^*^ | Difference  (w5-w1) | *Z(p)* ^*^ |
| --- | --- | --- | --- | --- | --- | --- | --- | --- |
| VAS of pain | DS(n=23) | 44.87 ±17.02 | 19.91±21.62 | -0.82  (0.412) | 32.13±23.65 | -0.63  (0.527) | 12.22±22.08 | -2.59  (0.010) |
|  | placebo(n=23) | 44.04 ±16.49 | 24.78±12.83 |  | 29.17±16.26 |  | 4.39±10.71 |  |
| Degree of edema | DS(n=23) | 0.67 ±0.89 | 0.44±0.93 | -0.66  (0.512) | 0.45±0.83 | -0.09  (0.929) | 0.01±0.59 | -0.96  (0.338) |
|  | placebo(n=23) | 0.52 ±0.67 | 0.24±0.53 |  | 0.40±0.70 |  | 0.15±0.61 |  |
| TOTAL  EQ-5D-5L | DS(n=23) | 10.48 ±3.06 | 2.13±2.69 | -0.51  (0.609) | 3.78±4.22 | -0.48  (0.633) | 1.65±4.13 | -1.29  (0.198) |
|  | placebo(n=23) | 10.17 ±2.61 | 2.57±1.78 |  | 3.52±2.84 |  | 0.96±2.14 |  |
| TOTAL FAOS | DS(n=23) | 307.43±82.19 | 61.13±72.52 | -0.91  (0.362) | 122.66±107.34 | -0.41  (0.684) | 61.53±99.49 | -1.15  (0.249) |
|  | placebo(n=23) | 299.54±76.45 | 83.91±74.37 |  | 128.46±85.78 |  | 44.55±53.31 |  |
| FAOS  Symptom/Rigidity | DS(n=23) | 67.10 ±18.05 | 8.36 ±15.97 | -1.42  (0.155) | 20.16±21.87 | -0.08  (0.939) | 11.80±17.34 | -2.54  (0.011) |
|  | placebo(n=23) | 66.50 ±13.45 | 14.69±12.83 |  | 18.74±17.07 |  | 4.05 ±10.47 |  |
| FAOS  ache | DS(n=23) | 60.62 ±16.98 | 15.52±14.41 | -0.80  (0.426) | 29.29±22.83 | -1.01  (0.312) | 14.25±20.91 | -2.69  (0.007) |
|  | placebo(n=23) | 62.18 ±15.56 | 18.60±16.60 |  | 24.41±18.33 |  | 5.81 ±10.12 |  |
| FAOS  Function, everyday life | DS(n=23) | 74.41 ±17.87 | 10.30±19.60 | -1.28  (0.202) | 18.04±24.12 | -0.51  (0.613) | 7.74±21.28 | -0.46  (0.644) |
|  | placebo(n=23) | 72.04 ±15.84 | 16.05±14.60 |  | 22.00±15.22 |  | 5.95 ±8.39 |  |
| FAOS  Features,Sports/  Leisure | DS(n=23) | 49.67 ±24.78 | 14.02±24.53 | -0.67  (0.502) | 28.80±30.07 | -0.97  (0.332) | 14.78±28.30 | -0.07  (0.947) |
|  | placebo(n=23) | 43.93 ±24.30 | 21.51±22.78 |  | 36.94±24.98 |  | 15.43±20.72 |  |
| FAOS  Quality of life | DS(n=23) | 58.30 ±19.93 | 10.47±21.06 | -0.01  (0.991) | 23.41±22.95 | -0.31  (0.758) | 12.94±23.24 | -0.10  (0.924) |
|  | placebo(n=23) | 54.92 ±22.45 | 13.03±22.13 |  | 26.34±22.22 |  | 13.31±16.14 |  |

**Supplementary Table S 3. Comparison of changes in outcome measurements between patients who received Dangguixu-san and those who received placebo for grade I acute lateral ankle sprain (n=23).**

| Dependent  Variables | Group  (n) | Week 0  (M±SD) | Week 1  (M±SD) | Week 5  (M±SD) | Difference  (w1-w0) | *Z(p)** | Difference  (w5-w0) | *Z(p)** | Difference  (w5-w1) | *Z(p)** |
| --- | --- | --- | --- | --- | --- | --- | --- | --- | --- | --- |
| VAS of pain | DS  (n=12) | 39.33±14.51 | 19.50±11.70 | 16.67±21.46 | -19.83±15.02 | -0.31  (0.755) | -22.67±24.64 | -0.56  (0.577) | -2.83±20.66 | -0.32  (0.750) |
|  | placebo  (n=11) | 43.91±21.19 | 22.82±12.24 | 14.73±10.56 | -21.09±15.00 |  | -29.18±19.81 |  | -8.09±8.62 |  |
| Degree of edema | DS  (n=12) | 0.17±0.39 | 0.17±0.58 | 0.25±0.45 | 0.03±0.36 | -0.24  (0.812) | 0.03±0.45 | -0.79  (0.431) | 0.00±0.67 | -0.69  (0.488) |
|  | placebo  (n=11) | 0.09±0.54 | 0.09±0.30 | 0.00±0.00 | -0.06±0.24 |  | -0.08±0.25 |  | -0.02±0.26 |  |
| TOTAL  EQ-5D-5L | DS  (n=12) | 9.33±1.78 | 7.08±1.56 | 7.50±3.66 | -2.25±1.82 | -0.84  (0.399) | -1.83±3.90 | -0.77  (0.439) | 0.42±4.10 | -0.51  (0.609) |
|  | placebo  (n=11) | 10.73±2.90 | 7.73±1.49 | 7.18±1.89 | -3.00±2.10 |  | -3.55±3.83 |  | -0.55±2.62 |  |
| TOTAL FAOS | DS  (n=12) | 349.28±60.85 | 393.40±51.89 | 403.23±98.09 | 44.13±52.35 | -0.19  (0.854) | 53.95±86.99 | -1.05  (0.295) | 9.83±100.83 | -0.86  (0.389) |
|  | placebo  (n=11) | 314.15±88.63 | 376.45±62.75 | 410.71±63.41 | 62.31±84.87 |  | 96.56±106.41 |  | 34.25±60.46 |  |
| FAOS  Symptom/  Rigidity | DS  (n=12) | 74.09±13.29 | 76.76±12.52 | 81.53±19.82 | 2.67±15.17 | -1.18  (0.240) | 7.43±18.11 | -0.96  (0.339) | 4.77±20.62 | -0.74  (0.459) |
|  | placebo  (n=11) | 68.93±15.98 | 78.55±13.07 | 80.83±16.85 | 9.62±15.00 |  | 11.90±21.57 |  | 2.28±11.89 |  |
| FAOS  ache | DS  (n=12) | 68.50±14.71 | 81.69±11.02 | 85.63±17.86 | 13.18±13.84 | -0.01  (0.999) | 17.13±21.40 | -0.09  (0.926) | 3.94±22.49 | -0.52  (0.600) |
|  | placebo  (n=11) | 64.91±19.28 | 78.77±9.25 | 83.58±14.67 | 13.89±18.48 |  | 18.70±23.99 |  | 4.81±12.24 |  |
| FAOS  Function, everyday life | DS  (n=12) | 79.99±12.75 | 88.71±12.11 | 86.38±18.66 | 8.72±8.21 | -0.06  (0.951) | 6.39±21.65 | -0.62  (0.538) | -2.33±19.39 | -1.05  (0.294) |
|  | placebo  (n=11) | 77.36±16.11 | 86.87±9.56 | 92.10±8.46 | 9.51±13.56 |  | 14.74±15.07 |  | 5.23±9.49 |  |
| FAOS  Features,  Sports/  Leisure | DS  (n=12) | 63.13±16.69 | 71.25±18.96 | 73.33±24.53 | 8.13±22.34 | -0.62  (0.537) | 10.21±21.70 | -1.45  (0.146) | 2.08±25.36 | -0.81  (0.419) |
|  | placebo  (n=11) | 51.85±23.24 | 68.64±13.98 | 78.64±12.27 | 16.79±23.46 |  | 26.79±28.01 |  | 10.00±18.44 |  |
| FAOS  Quality of life | DS  (n=12) | 63.55±20.10 | 75.00±10.99 | 76.35±22.10 | 11.45±18.64 | -0.28  (0.780) | 12.80±22.02 | -0.69  (0.488) | 1.35±20.67 | -1.59  (0.112) |
|  | placebo  (n=11) | 51.15±30.20 | 63.63±25.13 | 75.57±23.29 | 12.47±27.53 |  | 24.42±28.97 |  | 11.95±16.41 |  |

**Supplementary Table S 4. Comparison of outcome measurements between patients who received Dangguixu-san and those who received placebo for grade II acute lateral ankle sprain(n=23)**

| Dependent  Variables | Group  (n) | Week 0  (M±SD) | Week 1  (M±SD) | Week 5  (M±SD) | Difference  (w1-w0) | *Z(p)** | Difference  (w5-w0) | *Z(p)** | Difference  (w5-w1) | *Z(p)** |
| --- | --- | --- | --- | --- | --- | --- | --- | --- | --- | --- |
| VAS of pain | DS  (n=11) | 50.91±18.14 | 30.91±22.56 | 8.45±5.65 | -20.00±27.93 | -0.71  (0.479) | -42.45±18.38 | -1.54  (0.123) | -22.45±19.54 | -3.03  (0.002) |
|  | placebo  (n=12) | 44.17±11.65 | 16.00±15.47 | 15.00±15.08 | -28.17±9.93 |  | -29.17±13.11 |  | -1.00±11.65 |  |
| Degree of edema | DS  (n=11) | 1.36±1.03 | 0.27±0.47 | 0.36±0.50 | -0.95±1.10 | -1.31  (0.189) | -0.98±0.83 | -0.85  (0.396) | 0.03±0.51 | -0.72  (0.473) |
|  | placebo  (n=12) | 1.08±0.79 | 0.50±0.67 | 0.25±0.62 | -0.41±0.67 |  | -0.68±0.86 |  | -0.28±0.81 |  |
| TOTAL  EQ-5D-5L | DS  (n=11) | 11.73±3.72 | 9.73±3.35 | 5.82±0.75 | -2.00±3.49 | -0.88  (0.880) | -5.91±3.59 | -1.85  (0.065) | -3.91±2.88 | -2.66  (0.008) |
|  | placebo  (n=12) | 9.67±2.31 | 7.50±2.15 | 6.17±1.40 | -2.17±1.40 |  | -3.50±1.68 |  | -1.33±1.61 |  |
| TOTAL  FAOS | DS  (n=11) | 261.77±79.97 | 341.45±88.16 | 459.39±40.28 | 79.67±88.48 | -0.31  (0.758) | 197.62±71.97 | -1.23  (0.218) | 117.95±62.34 | -2.59  (0.010) |
|  | placebo  (n=12) | 286.15±64.35 | 389.86±72.46 | 443.85±52.68 | 103.71±60.16 |  | 157.70±49.73 |  | 53.99±46.46 |  |
| FAOS  Symptom/Rigidity | DS  (n=11) | 59.46±19.99 | 74.03±12.70 | 93.50±8.27 | 14.56±15.06 | -0.62  (0.534) | 34.04±16.88 | -1.33  (0.184) | 19.47±8.36 | -2.97  (0.003) |
|  | placebo  (n=12) | 64.28±10.87 | 83.63±8.54 | 89.29±9.87 | 19.34±8.67 |  | 25.01±8.35 |  | 5.67±9.20 |  |
| FAOS  ache | DS  (n=11) | 52.00±15.63 | 69.69±14.41 | 95.19±5.28 | 17.68±14.67 | -1.05  (0.294) | 43.18±14.36 | -2.35  (0.019) | 25.50±11.79 | -3.49  (<0.001) |
|  | placebo  (n=12) | 59.67±11.50 | 82.63±16.29 | 89.35±10.51 | 22.92±14.08 |  | 29.64±9.29 |  | 6.73±8.16 |  |
| FAOS  Function,  Everyday life | DS  (n=11) | 68.32±21.10 | 80.35±18.43 | 99.06±1.18 | 12.03±27.66 | -1.14  (0.254) | 30.75±20.61 | -0.09  (0.926) | 18.72±18.13 | -2.07  (0.039) |
|  | placebo  (n=12) | 67.16±14.54 | 89.21±10.89 | 95.83±6.57 | 22.05±13.31 |  | 28.67±12.47 |  | 6.62±7.61 |  |
| FAOS  Features,  Sports/Leisure | DS  (n=11) | 35.00±24.29 | 62.18±25.48 | 84.09±20.47 | 27.18±22.64 | -0.62  (0.536) | 49.09±24.58 | -0.62  (0.535) | 21.91±21.50 | -0.40  (0.687) |
|  | placebo  (n=12) | 36.67±23.87 | 62.50±28.16 | 82.92±18.88 | 25.83±22.24 |  | 46.25±18.36 |  | 20.42±22.20 |  |
| FAOS  Quality of life | DS  (n=11) | 52.57±18.98 | 61.97±21.73 | 87.56±14.21 | 9.40±24.32 | -0.15  (0.877) | 34.99±18.51 | -0.96  (0.338) | 25.59±19.52 | -1.70  (0.089) |
|  | placebo  (n=12) | 58.37±12.31 | 71.91±18.18 | 86.47±14.06 | 13.54±17.03 |  | 28.10±14.73 |  | 14.56±16.51 |  |

**Supplementary Table S 5. Comparison of change in clinical laboratory parameters between patients who received Dangguixu-san and those who received placebo for acute lateral ankle sprain (n=23 each).**

| Dependent  Variables | | | Week 0  (M±SD) | Week 1  (M±SD) | Difference  (w1-w0) | Z | p |
| --- | --- | --- | --- | --- | --- | --- | --- |
| CBC | WBC | DS (n=23) | 6.58±1.67 | 6.40±1.59 | -0.18±1.45 | -0.84 | 0.404 |
|  |  | Placebo (n=23) | 6.57±1.87 | 5.91±1.77 | -0.66±1.06 |  |  |
|  | RBC | DS (n=23) | 4.45±0.47 | 4.46±0.51 | 0.02±0.23 | -1.09 | 0.302 |
|  |  | Placebo (n=23) | 4.24±0.53 | 4.31±0.51 | 0.07±0.25 |  |  |
|  | Hemoglobin | DS (n=23) | 13.51±1.27 | 13.52±1.50 | 0.00±0.66 | -0.30 | 0.766 |
|  |  | Placebo (n=23) | 12.93±1.65 | 12.95±1.60 | 0.02±0.72 |  |  |
|  | Hematocrit | DS (n=23) | 39.64±3.67 | 39.88±4.32 | 0.24±1.95 | -0.86 | 0.391 |
|  |  | Placebo (n=23) | 37.39±4.76 | 38.05±4.42 | 0.66±2.19 |  |  |
|  | Platelet | DS (n=23) | 238.91±73.83 | 233.00±67.19 | -5.91±33.38 | -0.13 | 0.895 |
|  |  | Placebo (n=23) | 225.87±45.70 | 226.48±42.27 | 0.61±20.46 |  |  |
| Biochemistry | ALP | DS (n=23) | 104.96±63.08 | 102.61±58.76 | -2.35±12.23 | -0.30 | 0.767 |
|  |  | Placebo (n=23) | 99.26±46.63 | 98.17±48.06 | -1.09±14.94 |  |  |
|  | AST(SGOT) | DS (n=23) | 22.35±5.36 | 22.04±6.01 | -0.30±3.08 | -0.37 | 0.715 |
|  |  | Placebo (n=23) | 23.83±6.74 | 23.78±8.23 | -0.04±5.09 |  |  |
|  | ALT(SGPT) | DS (n=23) | 24.65±11.58 | 23.00±10.44 | -1.65±4.75 | -1.99 | 0.047 |
|  |  | Placebo (n=23) | 20.61±10.92 | 21.96±11.93 | 1.35±4.25 |  |  |
|  | T-Cholesterol | DS (n=23) | 195.43±48.77 | 191.39±44.71 | -4.04±22.16 | -0.51 | 0.613 |
|  |  | Placebo (n=23) | 194.35±35.10 | 193.22±27.40 | -1.13±25.50 |  |  |
|  | T-protein | DS (n=23) | 11.37±20.63 | 6.61±1.47 | -4.76±20.96 | -1.12 | 0.261 |
|  |  | Placebo (n=23) | 7.03±0.41 | 6.96±0.68 | -0.08±0.50 |  |  |
|  | Albumin | DS (n=23) | 4.30±0.43 | 4.23±0.48 | -0.07±0.36 | -0.68 | 0.499 |
|  |  | Placebo (n=23) | 4.39±0.39 | 5.70±6.41 | 1.31±6.50 |  |  |
|  | BUN | DS (n=23) | 13.23±3.43 | 13.21±5.16 | -0.02±3.18 | -0.22 | 0.826 |
|  |  | Placebo (n=23) | 14.08±3.83 | 13.32±4.04 | -0.77±3.41 |  |  |
|  | Cr | DS (n=23) | 0.82±0.21 | 0.80±0.21 | -0.02±0.15 | -2.05 | 0.040 |
|  |  | Placebo (n=23) | 0.78±0.17 | 0.84±0.19 | 0.07±0.14 |  |  |
|  | Glucose | DS (n=23) | 121.78±50.12 | 112.65±30.25 | -9.13±39.33 | -0.56 | 0.575 |
|  |  | Placebo (n=23) | 98.35±18.77 | 95.32±26.39 | -3.03±28.68 |  |  |
|  | gamma-GT | DS (n=23) | 30.22±21.88 | 26.58±20.02 | -3.64±9.02 | -1.79 | 0.073 |
|  |  | Placebo (n=23) | 20.39±12.64 | 21.13±14.06 | 0.74±4.04 |  |  |
|  | T-bilirubin | DS (n=23) | 0.70±0.34 | 0.71±0.32 | 0.01±0.21 | -0.01 | 0.999 |
|  |  | Placebo (n=23) | 0.65±0.24 | 0.67±0.29 | 0.02±0.20 |  |  |
|  | TG | DS (n=23) | 166.22±129.40 | 184.13±121.59 | 17.91±61.30 | -1.56 | 0.119 |
|  |  | Placebo (n=23) | 140.04±72.37 | 119.35±73.06 | -20.70±77.27 |  |  |
|  | HDL-C | DS (n=23) | 50.61±12.67 | 50.20±12.11 | -0.42±5.18 | -0.39 | 0.701 |
|  |  | Placebo (n=23) | 57.50±11.55 | 59.92±14.32 | 2.42±12.70 |  |  |
|  | LDL-C | DS (n=23) | 113.65±30.96 | 111.00±31.58 | -2.65±15.38 | -1.03 | 0.302 |
|  |  | Placebo (n=23) | 110.00±27.32 | 112.87±24.04 | 2.87±16.03 |  |  |
|  | Na | DS (n=23) | 183.61±206.98 | 140.78±2.00 | -42.83±206.93 | -0.21 | 0.833 |
|  |  | Placebo (n=23) | 140.70±2.08 | 141.43±2.41 | 0.74±2.18 |  |  |
|  | Cl | DS (n=23) | 104.65±2.53 | 105.26±2.83 | 0.61±2.31 | -0.26 | 0.795 |
|  |  | Placebo (n=23) | 104.43±1.73 | 142.87±205.58 | 38.44±205.28 |  |  |
|  | K | DS (n=23) | 4.28±0.36 | 4.19±0.37 | -0.09±0.41 | -0.31 | 0.757 |
|  |  | Placebo (n=23) | 4.17±0.40 | 8.46±21.05 | 4.30±21.02 |  |  |
| Urine  analysis | SG | DS (n=23) | 1.02±0.01 | 1.43±1.93 | 0.40±1.93 | 0.00 | 1.00 |
|  |  | Placebo (n=23) | 1.02±0.01 | 1.02±0.01 | -0.00±0.01 | -1.71 | 0.087 |
|  | PH | DS (n=23) | 5.63±0.73 | 5.98±1.15 | 0.35±1.16 | -1.30 | 0.193 |
|  |  | Placebo (n=23) | 6.15±0.98 | 6.52±1.17 | 0.37±1.06 | -1.59 | 0.113 |
|  | Nitrite  (negative) | DS (n=23) | 23(100.0%) | 23(100.0%) | 0(0.0%) | -1.00 | 0.32 |
|  |  | Placebo (n=23) | 22(95.7%) | 23(100.0%) | 1(0.43%) | 0.00 | 1.00 |
|  | Protein  (negative) | DS (n=23) | 20(87.0%) | 21(91.3%) | 1(0.43%) | -0.58 | 0.564 |
|  |  | Placebo (n=23) | 21(91.3%) | 23(100.0%) | 2(0.87%) | -1.41 | 0.157 |
|  | Glucose  (negative) | DS (n=23) | 22(95.7%) | 22(95.7%) | 0(0.0%) | 0.00 | 1.00 |
|  |  | Placebo (n=23) | 23(100.0%) | 23(100.0%) | 1(0.43%) | -1.00 | 0.32 |
|  | Ketone  (negative) | DS (n=23) | 22(95.7%) | 23(100.0%) | 1(0.43%) | -1.00 | 0.32 |
|  |  | Placebo (n=23) | 24(100%) | 23(100.0%) | 0(0.0%) | 0.00 | 1.00 |
|  | Urobilinogen  (negative) | DS (n=23) | 10(43.5%) | 10(43.5%) | 0(0.0%) | 0.00 | 1.00 |
|  |  | Placebo (n=23) | 9(39.1%) | 9(39.1%) | 0(0.0%) | 0.00 | 1.00 |
|  | Bilirubin  (negative) | DS (n=23) | 23(100.0%) | 23(100.0%) | 0(0.0%) | 0.00 | 1.00 |
|  |  | Placebo (n=23) | 23(100.0%) | 23(100.0%) | 0(0.0%) | 0.00 | 1.00 |
|  | WBC  (Microscopic)(0~4) | DS (n=23) | 20(87.0%) | 22(95.7%) | 2(0.87%) | -1.00 | 0.32 |
|  |  | Placebo (n=23) | 20(87.0%) | 20(87.0%) | 0(0.0%) | 0.00 | 1.00 |
|  | RBC  (Microscopic)  (0~4) | DS (n=23) | 21(91.3%) | 22(95.7%) | 1(0.43%) | -0.58 | 0.564 |
|  |  | Placebo (n=23) | 21(91.3%) | 23(100.0%) | 2(0.87%) | -1.41 | 0.157 |
